# Tools and best practices for retrotransposon analysis using high-throughput sequencing data

**DOI:** 10.1186/s13100-019-0192-1

**Published:** 2019-12-29

**Authors:** Aurélie Teissandier, Nicolas Servant, Emmanuel Barillot, Deborah Bourc’his

**Affiliations:** 1 Institut Curie, PSL Research University, 75005 Paris, France; 2 INSERM U900, 75005 Paris, France; 3 MINES ParisTech, PSL Research University, 75005 Paris, France; 4 INSERM U934, CNRS UMR 3215, 75005 Paris, France

**Keywords:** Retrotransposon, High-throughput sequencing, Data analysis, Mapping, Quantification

## Abstract

**Background:**

Sequencing technologies give access to a precise picture of the molecular mechanisms acting upon genome regulation. One of the biggest technical challenges with sequencing data is to map millions of reads to a reference genome. This problem is exacerbated when dealing with repetitive sequences such as transposable elements that occupy half of the mammalian genome mass. Sequenced reads coming from these regions introduce ambiguities in the mapping step. Therefore, applying dedicated parameters and algorithms has to be taken into consideration when transposable elements regulation is investigated with sequencing datasets.

**Results:**

Here, we used simulated reads on the mouse and human genomes to define the best parameters for aligning transposable element-derived reads on a reference genome. The efficiency of the most commonly used aligners was compared and we further evaluated how transposable element representation should be estimated using available methods. The mappability of the different transposon families in the mouse and the human genomes was calculated giving an overview into their evolution.

**Conclusions:**

Based on simulated data, we provided recommendations on the alignment and the quantification steps to be performed when transposon expression or regulation is studied, and identified the limits in detecting specific young transposon families of the mouse and human genomes. These principles may help the community to adopt standard procedures and raise awareness of the difficulties encountered in the study of transposable elements.

## Background

Transposable elements (TEs) comprise approximately half of the mammalian genomes [1]. Based on de novo repeat identification, it has been suggested that two-thirds of the human genome is in fact composed of repetitive elements [2].TEs are first classified according to their ability to invade the genome and their related molecular mechanisms. DNA transposons use a *cut-and-paste* mechanism where the element is excised and inserted into a new locus. Retrotransposons use an intermediate RNA template to insert into new genomic locations, in a *copy-and-paste* manner. These are classified into Long-Terminal Repeat (LTR) elements that are similar to retroviruses, and non-LTR elements. Non-LTR elements are more abundant compared to LTR elements and DNA transposons in mammalian genomes. The vast majority of TE insertions are incapable of mobilization, due to invalidating truncations, internal rearrangements or mutations. However, based on cell culture assays, it has been estimated that 80–100 L1HS elements are competent for retrotransposition in the human genome [3] and around 3000 L1 elements from the Tf, A and Gf subfamilies are potentially capable of retrotransposition in the mouse genome [4]. De novo insertions of TEs -mainly Alu, L1 and SVA non-LTR families- have been associated with more than 100 human diseases [5]. In reaction, cells have developed several restraining mechanisms against TE activity. At the transcriptional level, DNA methylation and repressive histone modifications block TE expression. In the cytoplasm, some restriction factors degrade retrotransposon RNAs. Other factors play a role in the nucleus by interfering with the DNA integration step [6].

The emergence of high-throughput sequencing technologies has allowed making tremendous progress in our understanding of the regulation and functional impact of TEs. However, the characterization of these elements remains computationally challenging, mainly due to their repetitiveness [6]. As they are not unique in the genome, repeated sequences create ambiguities in the alignment step, which can lead to misleading biological conclusions if inappropriate parameters are applied [7, 8]. Different algorithms have been developed for the purpose of mapping reads according to the sequencing application [9]. By default, most of these tools are parameterized to randomly report one genomic position among the set of possible alignments. Additional parameters or filters are implemented to keep uniquely mapped reads, to report all possible positions of reads or to return up to a given number of valid alignments. Benchmarkings of these methods have also been reported to compare their efficiency. Some of them investigated specific biological applications, such as Whole-Genome Bisulfite Sequencing (WGBS) [10] and RNA-seq [11] or specific sequencing platforms [12]. Schbath et al. assessed the power of tools to retrieve all the read occurences. However, their study relied on simulated short single-end reads of 40 bp without any insertions/deletions (indels). Hatem et al. investigated the effect of different mapping parameters such as number of mismatches, seed and read length, gapped vs ungapped alignment. Nevertheless, they did not investigate the power of the different algorithms to align TE-derived reads.

Some tools were developed to quantify TEs within sequencing data. TEtools uses TE annotation to create Bowtie2 index and performs mapping by reporting randomly one position [13, 14]. RepEnrich recommends performing the mapping with Bowtie to retrieve unique alignments [15, 16]. It enables quantifying unique reads emanating from specific families (referred to *repEnrich Unique* in this study) and the total number of reads, unique and multiple, mapped to each TE family (*repEnrich Total*). The *repEnrich Fractional* method counts reads that map to a single TE family and assigns multi-mapped reads to corresponding families using a fractional value 1/*n*, where *n* is the number of TE families the read maps to. SQuIRE [17] allows quantifying TE single copies and families performing the alignment with STAR [18] and using an iterative method to assign multi-mapped reads (*SQuIRE*). Finally, TEtranscripts [19] advises to generate BAM files with the STAR mapper, and performs TE quantification using only uniquely-mapped reads (*TEtranscripts Unique*), or using multi-mapped reads with an iterative method (*TEtranscripts Multiple*).

In this study, we propose to benchmark at once the efficiency of the most used aligners and available tools for TE quantification. Using simulated data with mouse and human genomes, Bowtie, Bowtie2, STAR, Novoalign (http://www.novocraft.com), BWA aln [20] and mem [21] alignment algorithms were compared. We also assessed the effect of using paired-end library compared to single-end library with TE-derived reads. Reporting unique reads, randomly one position and all possible locations were compared when TE abundance was estimated. In addition, TE quantification was compared to TE-simulated abundance using the most recent and used RepeatMasker-based tools, *TEtools*, *repEnrich, SQuIRE* and *TEtranscript*. Finally, the efficiency to map reads from each TE subfamily within the mouse and the human genome was computed and revealed the difficulties of accessing specific young TE families.

## Results

### Mapping based on STAR and PE libraries are highly recommended to align reads coming from transposable elements

**Fig. 1:**
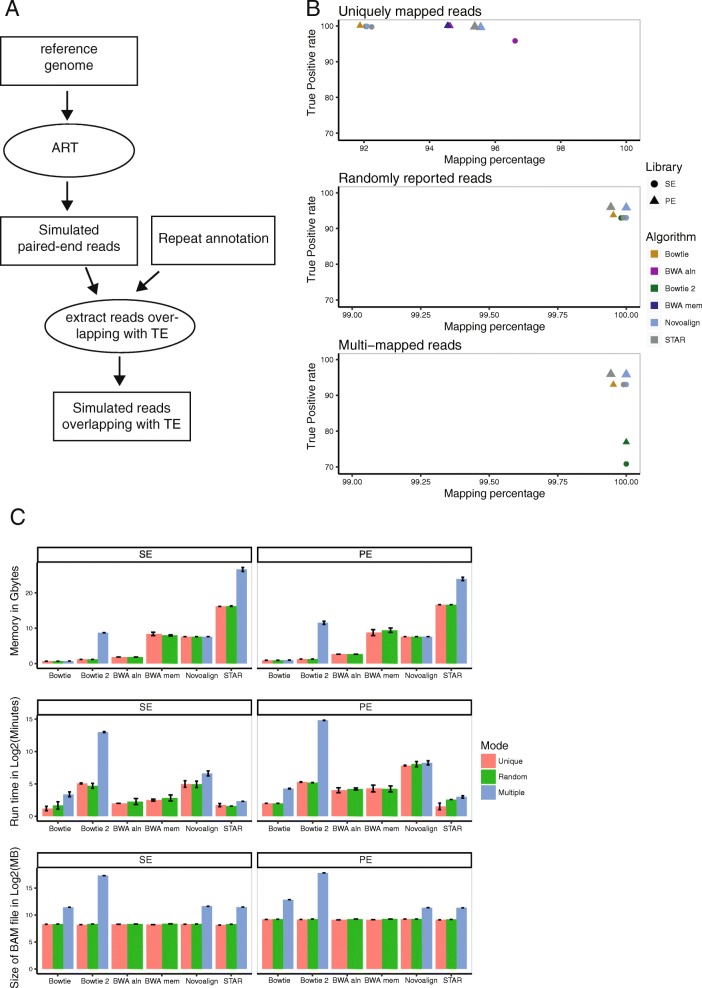
Comparison of mapper efficiency with mouse simulated data. **a** A diagram showing the method for the data simulation. The circles represent used tools and the rectangles correspond to files. **b** True Positive (TP) rate versus mapping percentage with chromosome 1 of the mouse genome. The dots are the average values of three independent simulated libraries. SE and PE refer to single end and paired end, respectively. **c** Use memory, run time and size of the BAM file with chromosome 1 of the mouse genome. The error bars correspond to standard deviation from three independent simulated libraries

To compare different mapping algorithms and their efficiency to align reads from repeated sequences, we relied on simulated data (Fig. 1a). Using a reference genome, 2x100bp paired-end reads were simulated with ART v2.5.8 [22] mimicking Illumina HiSeq 2500 technology (mean fragment size = 200 bp, standard deviation = 20 bp and technology-specific sequencing errors). Reads overlapping with *RepeatMasker* annotations were kept (Smit, R, & P, 2013–2015). Three independent datasets were simulated at a 10X coverage and aligned using Bowtie1, Bowtie2, BWA aln and mem algorithms, Novoalign and STAR. Only one end of the simulated fragments (single-end (SE) alignment) or both ends (paired-end (PE) alignment) were used, allowing us to compare the performance of both library types when TE-derived reads are aligned. Algorithms were run while enabling unique, randomly-reported or multi-mapped reads, except for BWA algorithms that do not give the possibility to return several hits per read. Reported alignments were compared to the simulated positions. When congruent, alignments were flagged as true-positive (TP) and weighted by the number of reported hits for the corresponding read in the multi-mapped mode. This approach allowed penalizing algorithms that report too many positions per read.

In Fig. 1b, TP rate and percentage of mapping were represented using the chromosome 1 of the mouse genome as the reference genome for the data simulation (Additional file 1: Figure S1A for the chromosome 1 of the human genome). In the top panel, uniquely-reported reads were considered. Around 92 and 95% of the reads were aligned in the SE and PE libraries respectively, highlighting the importance of using PE library to increase the uniqueness of fragments derived from transposon sequences. Conversely, Bowtie1 is the only tool which does not capitalize on the PE library to improve the mapping results. Some uniquely-mapped reads with SE library were not anymore mapped using paired-end information because the second read of the pair had different valid alignments.

Bowtie2, BWA mem and aln algorithms do not allow reporting uniquely mapped reads with defined parameters. Post-mapping filtering is therefore required. In this case, these mappers had the same performance with both SE and PE libraries compared to STAR and Novoalign (Fig. 1b and Additional file 1: Figure S1A and Tables 1 and 2).

**Table 1:** Statistics for the different mappers with mouse chromosome 1 simulation data

Algorithm	Library	Mode	Mapping percentage	True Positive rate	Memory in gbytes	Running Time in minutes	Output size in Mbytes
bowtie	PE	unique	91.87823	99.97913	0.92	3.00	583.36
bowtie	SE	unique	92.05224	99.92287	0.69	1.33	311.38
bowtie2	PE	unique	94.57886	99.93802	1.28	38.00	572.58
bowtie2	SE	unique	92.08282	99.84845	1.18	32.67	294.64
Bwa aln	PE	unique	94.62602	99.88782	2.66	15.67	553.86
Bwa aln	SE	unique	96.60879	95.82612	1.85	3.00	310.30
Bwa mem	PE	unique	94.54763	99.95728	8.77	19.33	563.50
Bwa mem	SE	unique	92.08548	99.89624	8.40	4.67	299.76
novoalign	PE	unique	95.55760	99.61473	7.62	226.33	609.08
novoalign	SE	unique	92.08982	99.92307	7.61	31.67	315.96
STAR	PE	unique	95.37882	99.80753	16.67	2.00	553.24
STAR	SE	unique	92.23340	99.73004	16.18	2.33	285.06
bowtie	PE	random	99.95300	93.67212	0.93	3.00	596.75
bowtie	SE	random	99.99001	93.04126	0.69	2.33	317.67
bowtie2	PE	random	99.99991	95.89737	1.28	35.67	607.86
bowtie2	SE	random	99.98093	92.97406	1.18	25.67	324.26
Bwa aln	PE	random	99.99998	95.94218	2.66	17.67	604.39
Bwa aln	SE	random	99.99801	93.01531	1.85	4.00	322.33
Bwa mem	PE	random	99.99998	95.94068	9.42	18.33	612.39
Bwa mem	SE	random	99.99998	93.01096	7.96	6.33	329.82
novoalign	PE	random	99.99998	95.84899	7.62	272.00	616.78
novoalign	SE	random	99.99989	93.03697	7.61	30.67	322.72
STAR	PE	random	99.94380	95.93094	16.67	5.00	583.02
STAR	SE	random	99.99024	93.01921	16.26	2.00	314.19
bowtie	PE	multi	99.95300	92.89719	0.98	18.33	7289.52
bowtie	SE	multi	99.99001	93.01711	0.71	9.67	2747.64
bowtie2	PE	multi	99.99998	76.80653	11.53	28658.67	228148.51
bowtie2	SE	multi	99.99998	70.81391	8.74	8205.33	161697.48
novoalign	PE	multi	99.99998	95.85903	7.62	307.67	2627.41
novoalign	SE	multi	99.99989	93.03718	7.61	99.00	3176.37
STAR	PE	multi	99.94380	95.93265	23.95	7.00	2575.59
STAR	SE	multi	99.99024	93.02143	26.64	4.00	2831.57

Values correspond to the average values of three independent simulated libraries with a 10X coverage. SE and PE refer to single end and paired end, respectively. Post-mapping filtering were applied for Bowtie2, Bwa mem and aln algorithms in order to extract uniquely-mapped reads

**Table 2:** Statistics for the different mappers with human chromosome 1 simulation data

Algorithm	Library	Mode	Mapping percentage	True Positive rate	Memory in gbytes	Running Time in minutes	Output size in Mbytes
bowtie	PE	unique	96.12725	99.99703	1.07	4.00	717.33
bowtie	SE	unique	96.26772	99.98760	0.80	1.67	381.52
bowtie2	PE	unique	97.58530	99.99163	1.42	36.00	720.57
bowtie2	SE	unique	96.25897	99.93671	1.33	25.33	375.46
Bwa aln	PE	unique	97.58600	99.99135	3.01	13.67	703.84
Bwa aln	SE	unique	98.40958	98.52603	2.18	6.33	381.22
Bwa mem	PE	unique	97.57669	99.99745	5.65	8.33	715.38
Bwa mem	SE	unique	96.28285	99.98096	5.45	4.67	379.88
novoalign	PE	unique	97.83211	99.99187	8.31	99.67	745.17
novoalign	SE	unique	96.28793	99.98755	8.31	21.00	385.94
STAR	PE	unique	97.79129	99.99166	18.12	2.33	693.70
STAR	SE	unique	96.29801	99.96226	17.71	1.00	363.12
bowtie	PE	random	99.95306	97.78786	1.07	4.00	722.46
bowtie	SE	random	99.98993	97.48616	0.80	2.33	383.45
bowtie2	PE	random	99.99967	98.68378	1.42	47.00	738.73
bowtie2	SE	random	99.97064	97.42861	1.33	35.67	391.06
Bwa aln	PE	random	99.99998	98.68727	3.01	13.67	733.20
Bwa aln	SE	random	99.99814	97.47704	2.18	7.33	387.77
Bwa mem	PE	random	99.99998	98.69222	6.05	9.33	744.88
Bwa mem	SE	random	99.99998	97.47710	5.26	3.00	397.18
novoalign	PE	random	99.99998	98.68797	8.31	100.67	748.47
novoalign	SE	random	99.99998	97.48725	8.31	27.67	388.19
STAR	PE	random	99.94355	98.68767	18.12	3.33	709.61
STAR	SE	random	99.99103	97.47578	17.70	2.00	378.46
bowtie	PE	multi	99.95306	97.41469	1.09	4.33	1032.87
bowtie	SE	multi	99.98993	97.47888	0.82	2.00	540.64
bowtie2	PE	multi	99.99998	85.55682	11.92	71150.67	81772.06
bowtie2	SE	multi	99.99998	77.59895	6.34	62006.33	123387.84
novoalign	PE	multi	99.99998	98.68698	8.31	83.67	800.39
novoalign	SE	multi	99.99998	97.48601	8.31	24.00	572.07
STAR	PE	multi	99.94355	98.69066	18.12	4.00	754.66
STAR	SE	multi	99.99103	97.47921	17.64	2.00	541.40

Values correspond to the average values of three independent simulated libraries with a 10X coverage. SE and PE refer to single end and paired end, respectively. Post-mapping filtering were applied for Bowtie2, Bwa mem and aln algorithms in order to extract uniquely-mapped reads

When randomly-reported and multi-mapped reads were allowed (middle and bottom panels, Fig 1b and Additional file 1: Figure S1A), the percentage of mapping increased close to 100%, leading to a decrease of TP rate around 93% for Bowtie1, 93% for the others in SE and 96% in PE. In addition, we also observed a big drop in Bowtie2 TP rate in the multi-mapped mode. Bowtie2 did not guarantee that the reported alignments are the best possible in terms of alignment score. Consequently, more alignments were reported, leading to a decrease of TP rate compared to other algorithms. As in unique mode, Bowtie1 was less efficient using PE library than SE library compared to Novoalign and STAR.

Computation time, BAM file size and memory usage were finally reported (Tables 1 and 2, Fig. 1c for mouse simulation and Additional file 1: Figure S1B for the human simulation) for all applied mappers and modes. The runtime measurement includes post-mapping filtering in the unique mode for bowtie2, BWA mem and aln algorithms. All algorithms required less than 10GB, except STAR which required 26GB at most. On the other hand, STAR was at least 15 times faster compared to Novoalign. Reporting all possible alignments per read increased at least four times the output size in PE mode compared to randomly-reported alignments for the mouse simulation. Output size of Bowtie2 in multi-mapped mode confirmed the fact that Bowtie2 reported too many alignments per read inducing a decrease of TP rate. In conclusion, STAR gave the best compromise in terms of mapping efficiency and accuracy, as well as computing time.

### Quantification of TE families: random and multiple counting methods give the best estimations

**Fig. 2:**
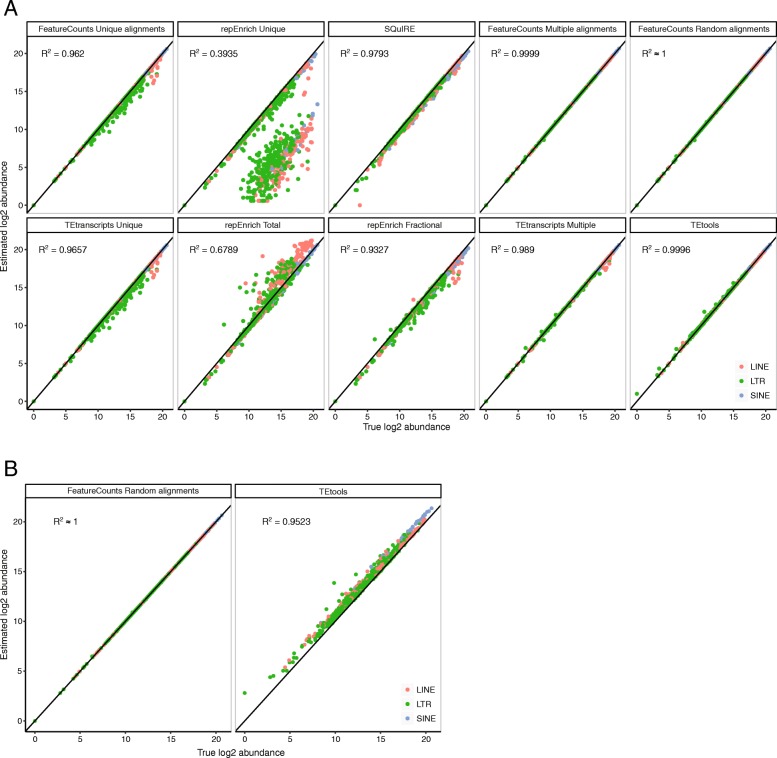
Comparison of the methods for the quantification of mouse retrotransposon families. **a** Comparison of the estimated abundance versus the true abundance for different quantification methods using mouse simulated TE-derived library. An R-squared value (R^2^) was calculated to evaluate the correlation of estimated values between simulated values **b** Comparison of the estimated abundance versus the true abundance for TEtools and when randomly reported reads are used for the TE quantification with FeatureCounts (*FeatureCounts Random alignments*). A PE genome-wide library (10X coverage) was simulated using the mouse genome with STAR for the mapping

Regarding its better performance, STAR was used as the mapping algorithm in subsequent quantification analyses. One library was simulated at a 10X coverage using the pipeline described previously with the human and mouse genomes as reference. The same mapping parameters than in previous analyses were applied for the human simulation. However, mapping parameters were adjusted (see Additional file 5) for the mouse simulation allowing higher number of multi-mapped reads, to account for the more complex TE content in this species. TE-estimated quantification from different developed methods was compared to simulated abundance. TE families were quantified using uniquely-mapped reads (referred to *FeatureCounts Unique alignments*), randomly-reported position (*FeatureCounts Random alignments*) and all valid alignments (*FeatureCounts Multiple alignments*). In the *FeatureCounts Multiple alignments*, alignments were weighted by the number of corresponding hits. Quantifications were performed using featureCounts [23]. In addition, repEnrich, TEtools, SQuIRE and TEtranscripts were evaluated using recommended parameters. TE-simulated abundance and estimated abundance were correlated for the different methods (Fig. 2a for mouse simulation and Additional file 3: Figure S2A for human simulation).

Methods using only unique reads (*FeatureCounts Unique alignments, repEnrich Unique, TEtranscripts Unique*) underestimated some TE families of all classes (LTR, LINEs and SINEs), with *repEnrich Unique* being the least accurate. In contrast, counting the total number of reads mapping to each TE family -as it is the case with *repEnrich Total-* induced an overestimation. On the other hand, weighting by the number of hits (*FeatureCounts Multiple alignments*) or reporting randomly one position (*TEtools* and *FeatureCounts Random alignments*) gave the most satisfactory TE estimation with a correlation close to 1. To test whether coverage could influence these results, we repeated the simulation with 5X, 10X, 25X, 50X and 100X coverage, focusing on specific TE families known to be potentially active (B2_Mm1a, IAPEz-int and L1MdA_I for the mouse genome and AluYa5, HERVK-int, L1HS and SVA_F for the human genome). Independently of the coverage depth, methods using unique reads (*FeatureCounts Unique alignments, repEnrich Unique, TEtranscripts Unique*) consistently underestimated TE families (Additional file 3: Figure S3A and B), while *FeatureCounts using random and multiple alignments* and *TEtools* gave the best estimation, confirming the 10X genome-wide simulation.

By proposing to map reads on TE annotations only, TEtools contrasts with other mapping methods that align reads genome-wide and then extract TE-derived reads only. However, because transposable elements represent only half of the mammalian genomes, we wanted to estimate whether TEtools could introduce some biases. New datasets were then simulated uniformly genome-wide, including non-repeated sequences, by generating PE libraries with a 10X coverage from mouse and human genomes. Compared to the *FeatureCounts Random alignments* (with STAR for the mapping), TEtools clearly introduced an overestimation of both LINE1 and LTR elements by forcing non-derived reads to map to TE sequences (Fig. 2b and Additional file 2: Figure S2B).

### Evolutionarily young families suffer from low percentage of mapping and low true positive rate

**Fig. 3:**
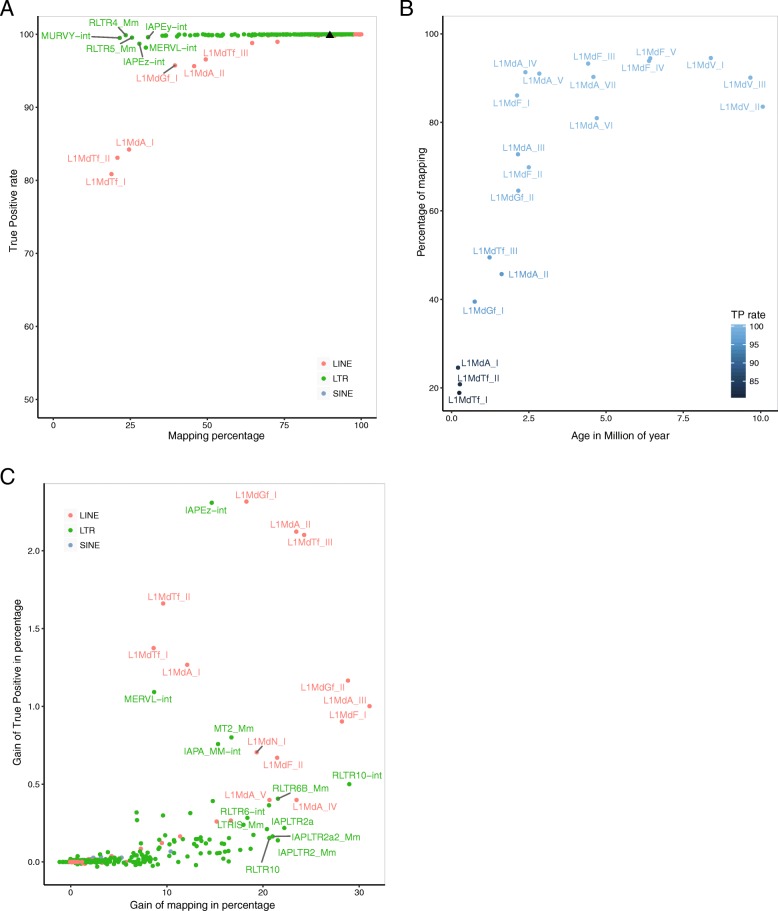
Mappability of the different mouse retrotransposon families. **a** True Positive (TP) rate versus mapping percentage per TE family using STAR and paired-end library with mouse simulated TE-derived reads. Black triangle represents the True Positive rate and percentage of mapping for the entire simulated library. **b** Mapping percentage versus age of L1Md families. Dot colors represent the True Positive (TP) rate. Ages are obtained from previously published divergence analysis study [24] **c** Gain of True Positive in percentage versus gain of mapping in percentage when PE library are used in comparison to SE library

Using PE library simulated on the mouse and human genomes, we found that 89.8 and 93.4% of the reads were uniquely mapped, respectively, with a TP rate of 99.9% (Fig. 3a and Additional file 4: Figure S4A). However, we noticed that some TE families displayed a lower mapping percentage. This was the case for the L1HS family –a recent human-specific L1 family- whereby 49% of simulated reads had 88% of TP rate upon unique mapping. In the mouse genome, 25 families had less than 50% of mapping when uniquely-reported reads were allowed, six of them being annotated in the LINE order. Using estimated evolutionary age of mouse and human LINE1 families [24, 25], we found that the youngest families were the ones with the lowest percentage of mapping and TP rate (Fig. 3b and Additional file 4: Figure S4B). These two metrics appear therefore as new classifiers to rank L1 subfamilies according to their age. The link between mappability and the age of L1 families was previously reported by Sexton and Han for the human genome [26]. The lower the age is, the lower mappability is as well.

Among the 25 mouse TE families with less than 50% of mapping, 19 were annotated as LTR retrotransposons, with representatives of the three different classes of LTR defined by their similarities to exogenous retroviruses [27]. In the ERV1 class, MURVY-int, its related LTR (RLTR5_MM) and RLTR4_MM (LTR flanking Murine Leukemia virus elements) had less than 25% of mapping. In the ERVK class, reads corresponding to the IAPEz-int annotation had 28% of mapping. This annotation represents the internal portion of IAPLTR1 elements, which are the young active elements from the IAP subtypes [28]. Finally, MERVL-int annotations, which represent active members of the ERVL class, had only 30% of mapping [29].

As depicted in Fig. 1b and Additional file 1: Figure S1A, using PE library improved the mapping step by producing a higher percentage of uniquely-mapped reads: more precisely, 6 and 2% of additional uniquely-mapped reads were gained in genome-wide mouse and human simulations, respectively. However, there was a strong inter-TE family variability in the improvement (Fig. 3c and Additional file 4: Figure S4C). Mouse L1MdGf_II, L1MdA_III and L1MdF_I (Fig. 3c) and human L1PA3 and L1PA2 (Additional file 4: Figure S4C) showed a 30% mapping gain when a PE library was used. The gain was slightly less satisfactory for the youngest LINE1 families compared to the slightly older families mentioned above, with human L1HS gaining 22% (Additional file 4: Figure S4C) and mouse L1MdTf_I, L1MdTf_II and L1MdA_I gaining 10% only on average (Fig. 3c). Similarly, in the human genome, mapping was improved by 20% or more when using PE over SE libraries for the youngest, hominoid-specific SVA subtypes (SVA_E and SVA_F) and the youngest subfamilies of the AluY type (AluYa5 and AluYb9)(Additional file 4: Figure S4C) [29–31]. These results demonstrate the importance of paired-end sequencing libraries, especially for the study of evolutionarily young TE families, provided that they are not completely identical in sequence.

## Discussion

Because of their repetitive nature, TE-derived sequences are complex to analyze. The objective of the present study was to provide objective guidelines for the analysis of transposable elements within high-throughput sequencing datasets.

### Sample and library preparation

At the beginning of a project, experimental design and sample preparation should be conceived in order to retrieve as much information as possible. Chhangawala et al (2015) already showed that single-end reads increased the number of multi-mapped reads. In contrast, paired-end reads lower the amount of multi-mapped reads and increase splicing event detection [32]. Our study confirms the importance of using paired-end library instead of single-end when analyzing TE-derived reads, especially for evolutionarily young families such as SVA_F, AluYb9 and L1HS in the case of human-based analyses. Read length is another parameter to take into consideration when TE-derived reads are sequenced. Chhangawala et al (2015) showed that longer reads increased the uniqueness of sequenced fragments. Longer fragment size should also help during the mapping step, because the chance for the sequenced fragment to fall into the boundaries or to cover a polymorphism will increase with the size of the fragment. As a result, the mappability of the given fragment should increase. However, having longer reads is a limitation of the Illumina technology. It is also a limiting factor in some applications, such as ChIP-seq, CUT&RUN and WGBS, where fragment size is determined by obligate fragmentation steps (sonication, micrococcal nuclease digestion or bisulfite-induced DNA degradation).

### Mapping

After quality control, read alignment against a reference genome is the first step in NGS analyses. Appropriate parameters and algorithms are needed to align as many TE-derived reads as possible. BWA algorithms (mem and aln) and bowtie2 have no defined parameter for retrieving uniquely mapped reads. In such case, post-mapping filtering has to be applied. In contrast, Novoalign, bowtie and STAR have dedicated parameters to report uniquely-mapped reads. However, bowtie does not capitalize on the information of paired-end reads. If a 5’end read -R1 read- is uniquely mapped and the corresponding 3’end read -R2 read- is a multi-mapper, bowtie discards the valid alignment from the R1 read. In contrast, Novoalign and STAR use the information from the R1 read and increase the percentage of mapping with paired-end library.

In the multiple-hit mode, Bowtie2 searches for up to k valid alignments per read, where k is a threshold given by the user (k was set to 5000 in this study). In Bowtie2’s reference manual, it is mentioned: “Bowtie 2 does not guarantee that the k alignments reported are the best possible in terms of alignment score” (http://bowtie-bio.sourceforge.net/bowtie2/manual.shtml). Other alignments with different alignment scores are reported in addition to the best alignment, which creates a low true positive rate and a bigger BAM file compared to STAR and Novoalign (Tables 1 and 2).

We found that reporting multi-mapped reads or reporting randomly one position increases the percentage of mapping close to 100% but at the cost of lower precision, which confirms previous results [11, 33]. Discarding multi-mapped reads is a real cost for evolutionary young families due to quasi-identical copies. However, these families are the ones that are mostly regulated in the genome, by repression histone marks and DNA methylation [34, 35]. As a conclusion, using multi-mapped reads or reporting randomly one position has to be done with caution to avoid discarding the most important information of the TE fraction of the genome.

As with the uniquely-mapped reads, STAR and Novoalign were the best compromise to report multi-mapped reads or a random valid alignment. However, Novoalign had a big disadvantage, its computing time, especially using PE reads. Starting with more than three millions of paired-end reads simulated from the mouse chromosome 1, Novoalign randomly aligned this set of reads in 4.5 h (Tables 1 and 2), while STAR completed the same task in 5 min. As the amount of sequenced reads and the number of projects with sequencing data are growing, fast algorithms are requested. This is why we recommend using STAR for the mapping step. Nevertheless, specific parameters have to be adapted for the study of transposable elements. This is especially important for young families that display a low mappability score. Unadapted parameters can mask relevant results or on the contrary, create incorrect conclusions. By default, STAR reports up to 10 alignments per read. The ENCODE project recommends to report up to 20 alignments per reads for long RNA sequencing pipeline. These guidelines are adapted for pseudogenes. In the case of TE studies and genomes with high TE content, these parameters have to be tuned (see Methods). A previous study based on ChIP-seq data estimated that a threshold of 10,000 positions per read is optimal in term of computing time and storage, without significant loss of sequence information (0.25% of reads eliminated on average) [35].

### Quantification of transposable elements

To highlight TE regulation, transposable element quantification is estimated and compared in different biological conditions. Dedicated methods have to be applied according to the parameters used during the alignment step. We demonstrated that quantification methods relying on uniquely-mapped reads underestimated the abundance of the youngest TE families, because of their low level of sequence diversity and consequently, low mapping performance.

When using reads with multiple hits, we found that reporting randomly one position or weighting multi-mapped reads with the number of hits give rise to the same estimation. However, reporting multi-hits is more consuming in terms of storage and time. In the case of mouse simulation, the output is five times bigger (500 Mbytes to 2500 Mbytes) when multi-hits are reported in comparison to the random mode. The increase in the rate and amount of sequencing data represents a high storage challenge for the community. Data analyses within TE studies has to be conducted with taking care of the amount of processed data. For this reason, we recommend to report randomly one position per read.

We also studied the specific case of TEtools*,* which quantifies TEs using randomly reported reads with Bowtie or Bowtie2. However, this tool considers a list of TE sequences extracted from a genome or manually annotated- as genomic references for the mapping. We showed that, in the case of available assembled genomes, performing the mapping onto the reference genome gives rise to a better estimation of TE quantity in comparison to the strategy applied by TEtools. Indeed, using only a part of the genome assembly introduces a bias in the alignment by forcing the mapping to this genome extract, the extent of which results from a combination of technology-specific sequencing errors and mismatch allowance in the alignment settings. Consequently, regions represented in this genome extract are overestimated. The method used by TEtools is analogous to a strategy where TE consensus sequences provided by RepBase are used for the mapping step [36]. Aligning reads against consensus sequences should also lead to an overestimation of the abundance of TEs; it adds moreover another confounding factor by allowing more mismatches. In the case of available assembly genomes, we therefore recommend to align reads with the reference genome and extract expression with FeatureCounts. Then, for RNA-seq analyses, gene quantification can be performed in the same time taking, advantage of only one step. Gene and transposon-based differential expression should be called in the same analysis, as it is done in *SQuIRE* and *TEtranscripts*.

### Transposable elements and their evolution

Human and mouse genomes are estimated to contain 48.5 and 41.8% of TEs, respectively. Interestingly, using genome-wide simulation on these species, we observed a higher mappability in the human genome compared to the mouse one. These differences likely reflect a more recent activity of certain TE families in the mouse genome, and therefore a higher proportion of sequence homology among TE copies. The overview we provide here on the TE-specific mappability rate should help researchers qualifying their conclusions made on specific families. For instance, in the mouse, using uniquely-mapped reads on L1 young families, IAPEz and MERVL families will undoubtedly induce an underestimation of their abundance in NGS datasets. We demonstrate and quantify here that significant improvement − 20 to 30% of mapping gain- can be obtained for these young TE families by using PE library. This is truly important, particularly in RNA-seq datasets, as these families are the ones that have more intact sequences, including at transcription factor binding sites, and therefore the potential for being transcribed.

## Conclusions

By comparing different available algorithms with simulated data generated onto the mouse and human genomes, we demonstrated the difficulty of analyzing evolutionarily young TE families. Improvements can nonetheless be gained if the following recommendations are followed:

1. paired-end library should be used to increase the uniqueness of sequenced fragments.
2. During the alignment step, STAR is the best compromise between efficiency and speed. Parameters have to be set according to the TE content.
3. Reporting randomly one position and using FeatureCounts to quantify TE families gives the best estimation values.
4. When TE annotation on an assembled genome is available, mapping and quantification should be done with the reference genome.
5. Evolutionarily young families suffer from low mappability rate and are severely underestimated if uniquely-mapped reads are reported.

## Methods

### Reconstruction of repeatMasker annotations

Transposon annotations were downloaded from the RepeatMasker website (Smit, AFA, Hubley, R & Green, P. *RepeatMasker Open-4.0*. 2013–2015 <http://www.repeatmasker.org>). As described in Bailly-Bechet et al., 2014, a dictionary was constructed for LTR retrotransposons that associated elements corresponding to the internal sequence and those corresponding to LTR sequences. With the latter and the RepeatMasker database, fragments of transposable elements corresponding to the same copy were merged if the distance between them is less than 1000 bp.

### Simulation data pipeline

2x100bp paired-end reads were simulated with ART v2.5.8 [22] mimicking Illumina HiSeq 2500 technologies (−m 200 –s 10). Simulated reads overlapping with reconstructed repeatMasker annotation were kept using Bedtools intersectBed v2.21.0.

### Mapping comparison

The following tools were used: Bowtie v1.0.0, Novoalign v3.2.11, STAR v2.5.2b, Bowtie2 v2.1.0, BWA aln v0.7.15, BWA mem v0.7.15. All the mappers were run with four threads (except for Novoalign that can be run with only 1 thread). Parameters used for the unique, random and multiple mode are detailed in Additional file 5.

### Quantification comparison

The following tools were compared. Command lines and parameters are detailed in Additional file 5.

#### repEnrich

as recommended, reads were first mapped with Bowtie v1.2 reporting unique alignments and retrieving multi-hits in fastq files (−m1 --max multimap.fastq). TE families were quantified using repEnrich v0.1.

#### TEtools

repeatMasker annotation was first extended 300 bp upstream and downstream in order to map reads located in the boundaries. TEtools v1.0.0 was used with Bowtie2 v2.2.4.

#### TEtranscripts

STAR v2.5.2b was used with the recommended parameters (−- outAnchorMultimapNmax 100 --outFilterMultimapNmax 100). TEtranscipts v1.5.1 was run using unique and multiple modes.

#### SQuIRE

To compare TE-estimated abundance with other tools, the same TE annotation was provided to the clean folder (−c option in SQuIRE count). SQuIRE v0.9.9.92 was run.

#### FeatureCounts unique, random and multiple alignments

featureCounts v1.5.1 was used with specific options (−s 0 -p). The option -M was used for random and multiple counting methods. In the multiple counting method, −-fraction option was also used in order to weight the counts for multi-mapped reads. Quantification of TE family was performed by summing all copies from each family.

## Supplementary information

**Additional file 1: Figure S1.** Comparison of mapper efficiency with human simulated data. (A) True Positive (TP) rate versus mapping percentage with chromosome 1 of the human genome. The dots are the average values of three independent simulated libraries. SE and PE refer to single end and paired end, respectively. (B) Use memory, run time and size of the BAM file with chromosome 1 of the human genome. The error bars correspond to standard deviation from three independent simulated libraries.

**Additional file 2: Figure S2.** Comparison of the methods for the quantification of human retrotransposon families. (A) Comparison of the estimated abundance versus the true abundance for different quantification methods using human simulated TE-derived library. An R-squared value (R2) was calculated to evaluate the correlation of estimated values between simulated values (B) Comparison of the estimated abundance versus the true abundance for TEtools and when randomly reported reads are used for the TE quantification with FeatureCounts (FeatureCounts Random alignments). A PE genome-wide library (10X coverage) was simulated using the human genome with STAR for the mapping.

**Additional file 3: Figure S3.** Impact of read depth in TE families quantification. (A) Estimated abundance for different quantification methods and true abundance (Simulated counts) using 5X, 10X, 25X, 50X and 100X coverage on specific mouse TE families. Only these TE families were used for the quantification. (B) Same as in A), with specific human TE families.

**Additional file 4: Figure S4.** Mappability of the different human retrotransposon families. (A) True Positive (TP) rate versus mapping percentage per TE family using STAR and paired126 library and human simulated TE-derived reads. Black triangle represents the True Positive rate and percentage of mapping for the entire simulated library (B) Mapping percentage versus age of L1Md families. Dot colors represent the True Positive (TP) rate. Ages are obtained from previously published divergence analysis study (25) (C) Gain of True Positive in percentage versus gain of mapping in percentage when PE library are used in comparison to SE library.

**Additional file 5.** Supplementary methods.

## Data Availability

Data sharing not applicable to this article as no datasets were generated or analysed during the current study.

## Abbreviations

ERV: Endogenous Retrovirus
LINE: Long INterspersed Element
LTR: Long Terminal Repeat
PE: Paired-End
SE: Single-End
SVA: SINE-R, VNTR, and *Alu*
TE: Transposable Element
TP: True Positive
