# Supplementary figures and images for: Tools and best practices for retrotransposon analysis using high-throughput sequencing data
Source: Mob DNA. 2019 Dec 29;10:52. doi: 10.1186/s13100-019-0192-1 (PMC6935493; doi:10.1186/s13100-019-0192-1)

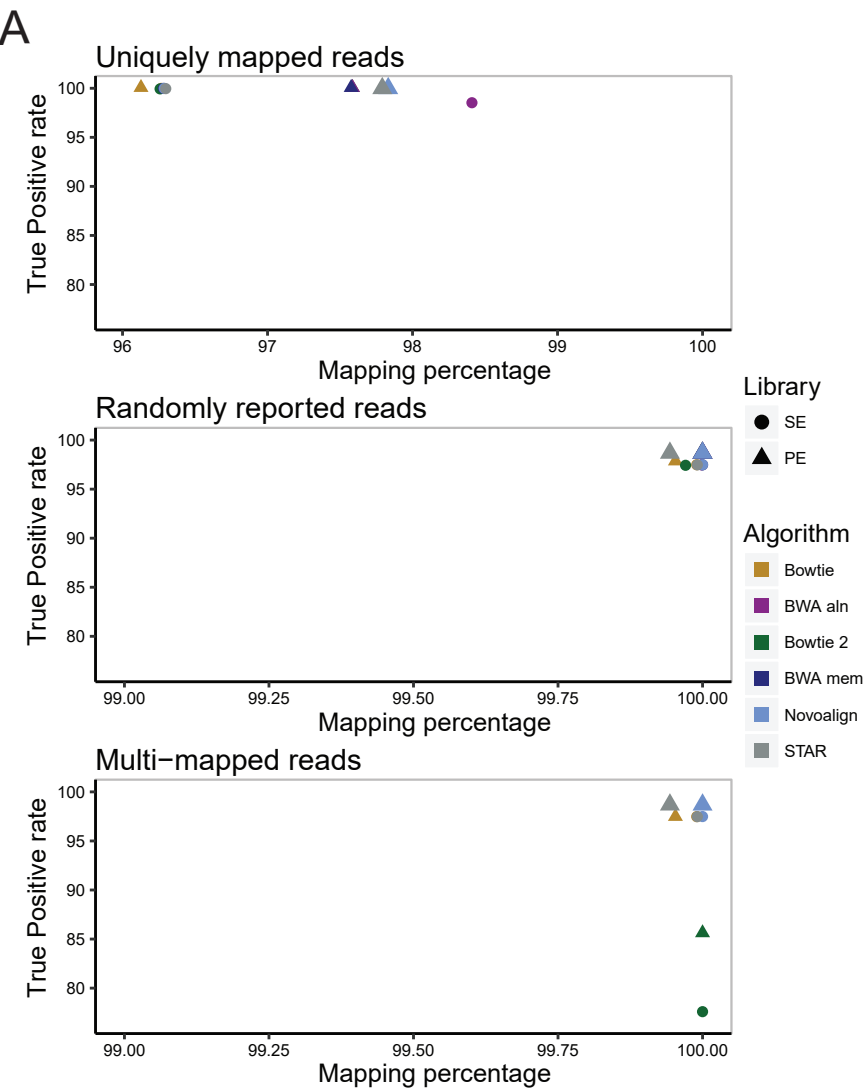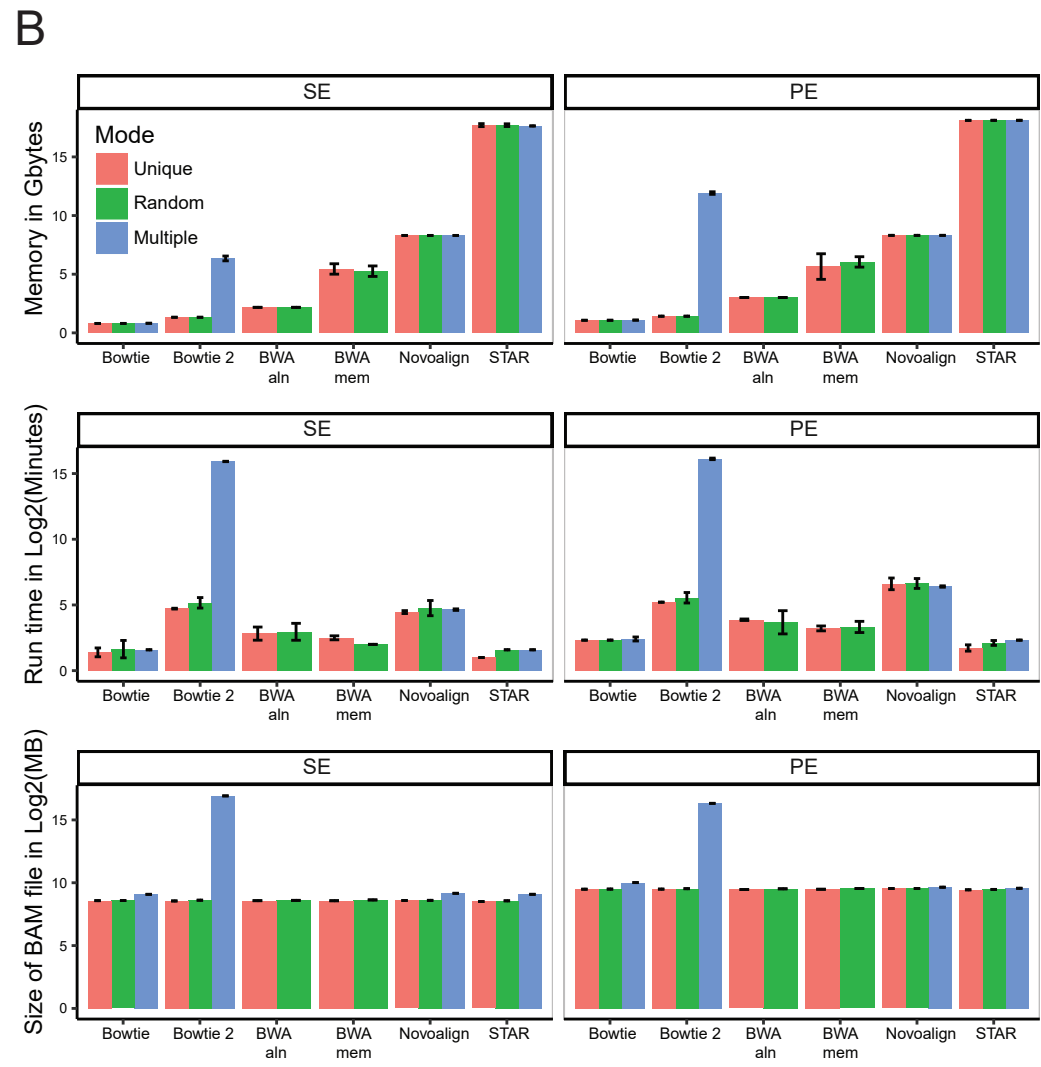

Supplement: Supplementary file 1 — Additional file 1: Figure S1. Comparison of mapper efficiency with human simulated data. (A) True Positive (TP) rate versus mapping percentage with chromosome 1 of the human genome. The dots are the average values of three independent simulated libraries. SE and PE refer to single end and paired end, respectively. (B) Use memory, run time and size of the BAM file with chromosome 1 of the human genome. The error bars correspond to standard deviation from three independent simulated libraries. [file 13100_2019_192_MOESM1_ESM.pdf]

A

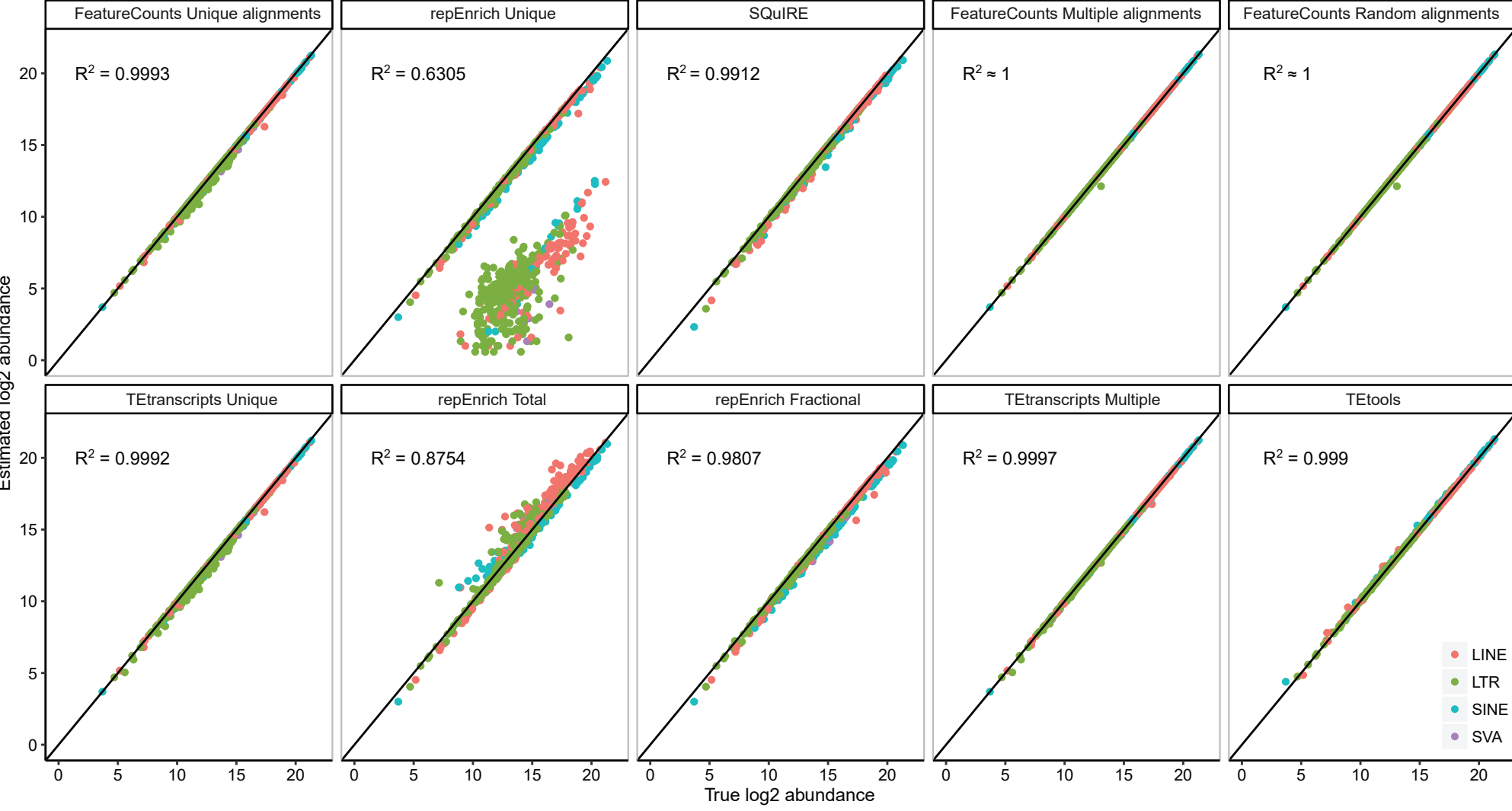

B

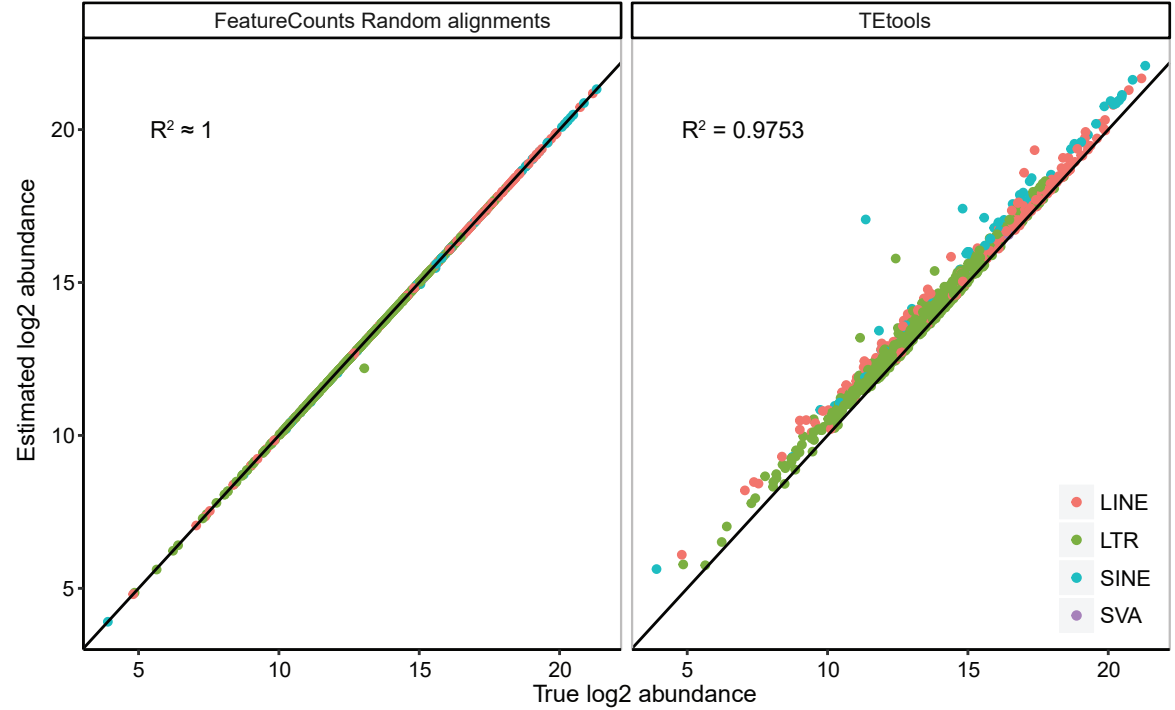

Supplement: Supplementary file 2 — Additional file 2: Figure S2. Comparison of the methods for the quantification of human retrotransposon families. (A) Comparison of the estimated abundance versus the true abundance for different quantification methods using human simulated TE-derived library. An R-squared value (R2) was calculated to evaluate the correlation of estimated values between simulated values (B) Comparison of the estimated abundance versus the true abundance for TEtools and when randomly reported reads are used for the TE quantification with FeatureCounts (FeatureCounts Random alignments). A PE genome-wide library (10X coverage) was simulated using the human genome with STAR for the mapping. [file 13100_2019_192_MOESM2_ESM.pdf]

A

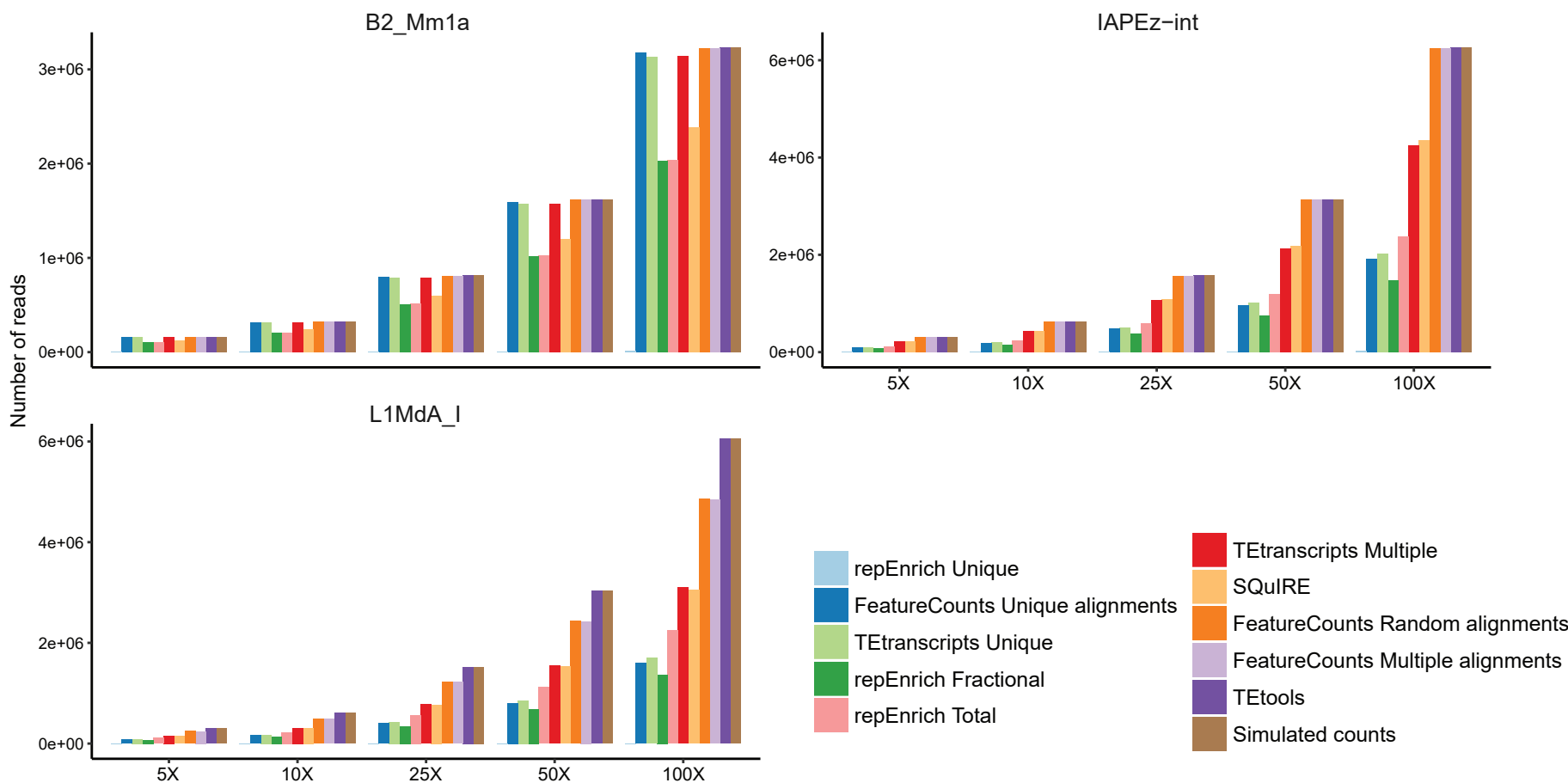

B

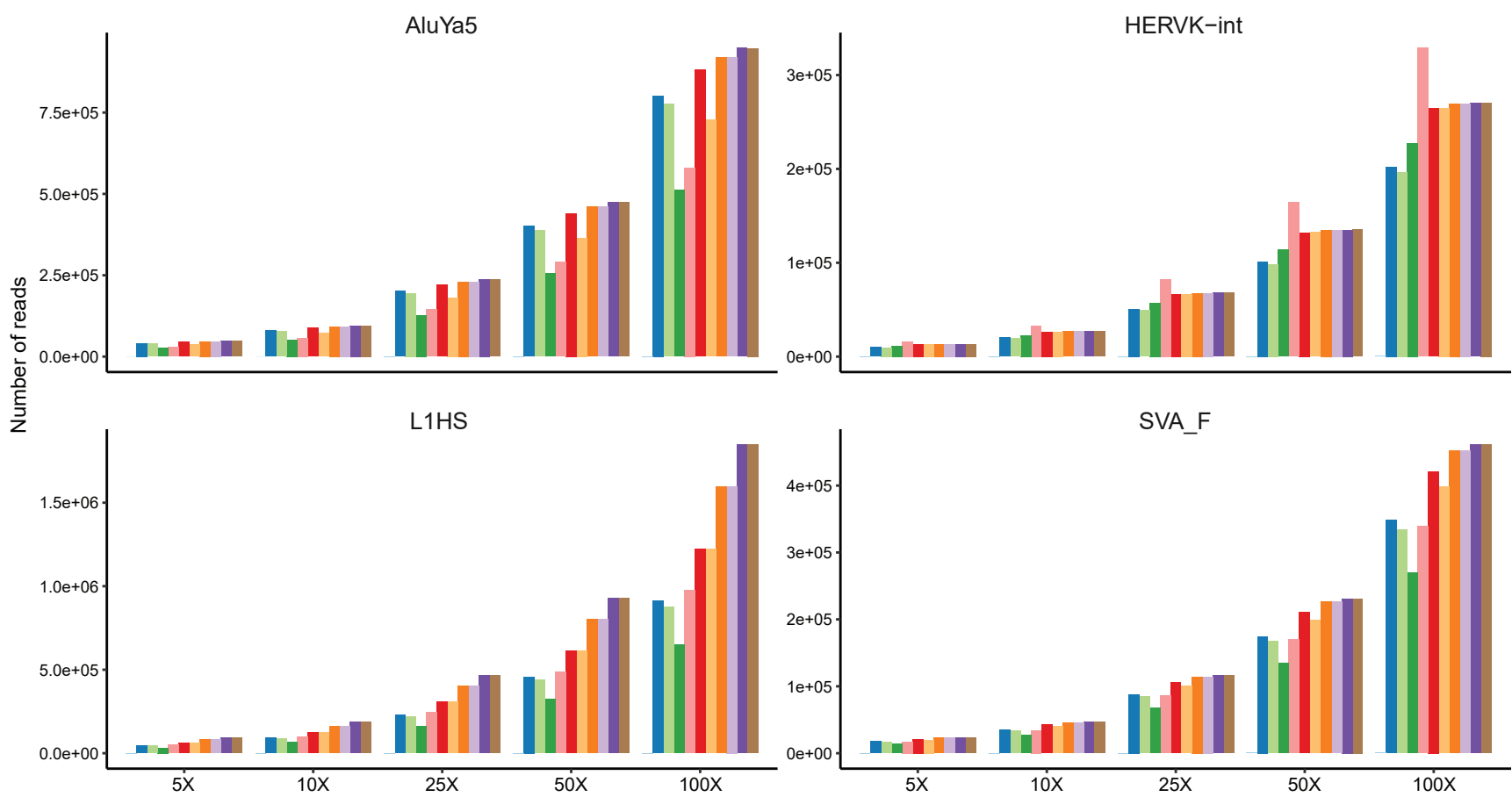

Supplement: Supplementary file 3 — Additional file 3: Figure S3. Impact of read depth in TE families quantification. (A) Estimated abundance for different quantification methods and true abundance (Simulated counts) using 5X, 10X, 25X, 50X and 100X coverage on specific mouse TE families. Only these TE families were used for the quantification. (B) Same as in A), with specific human TE families. [file 13100_2019_192_MOESM3_ESM.pdf]

A

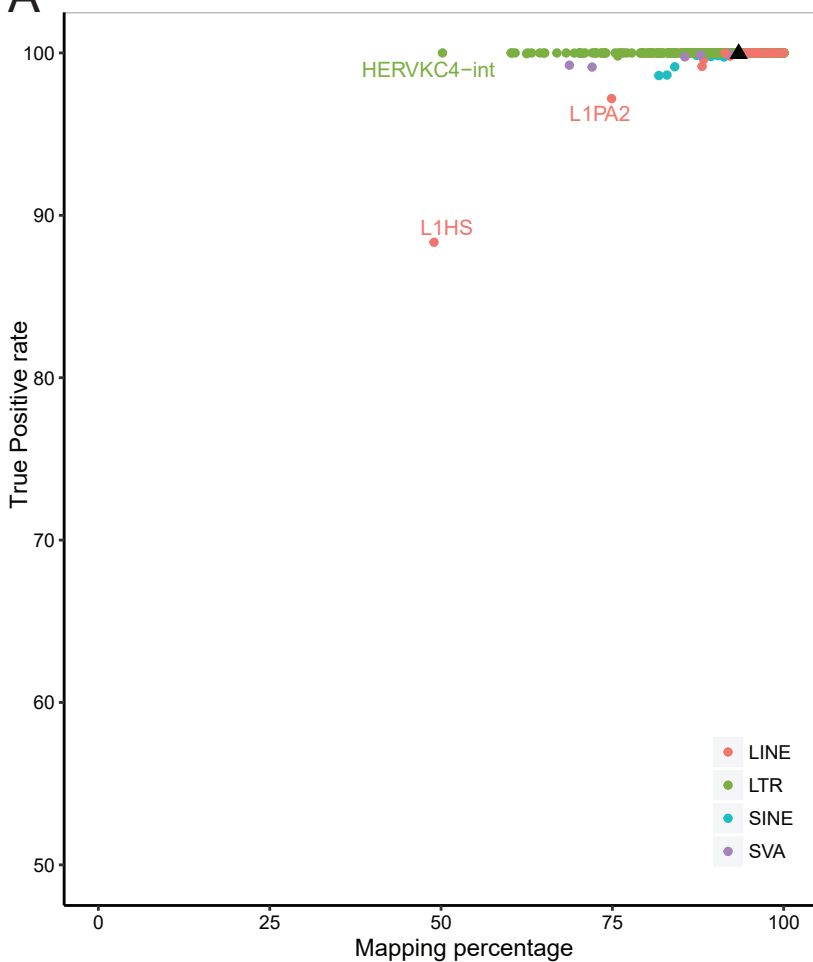

B

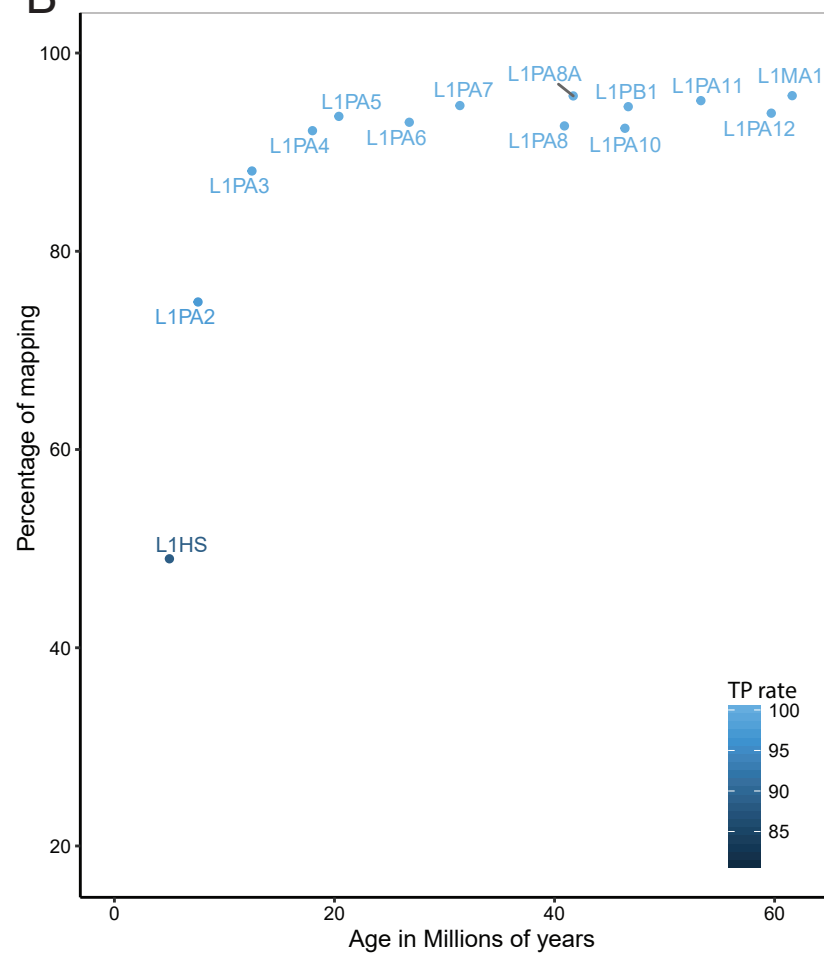

C

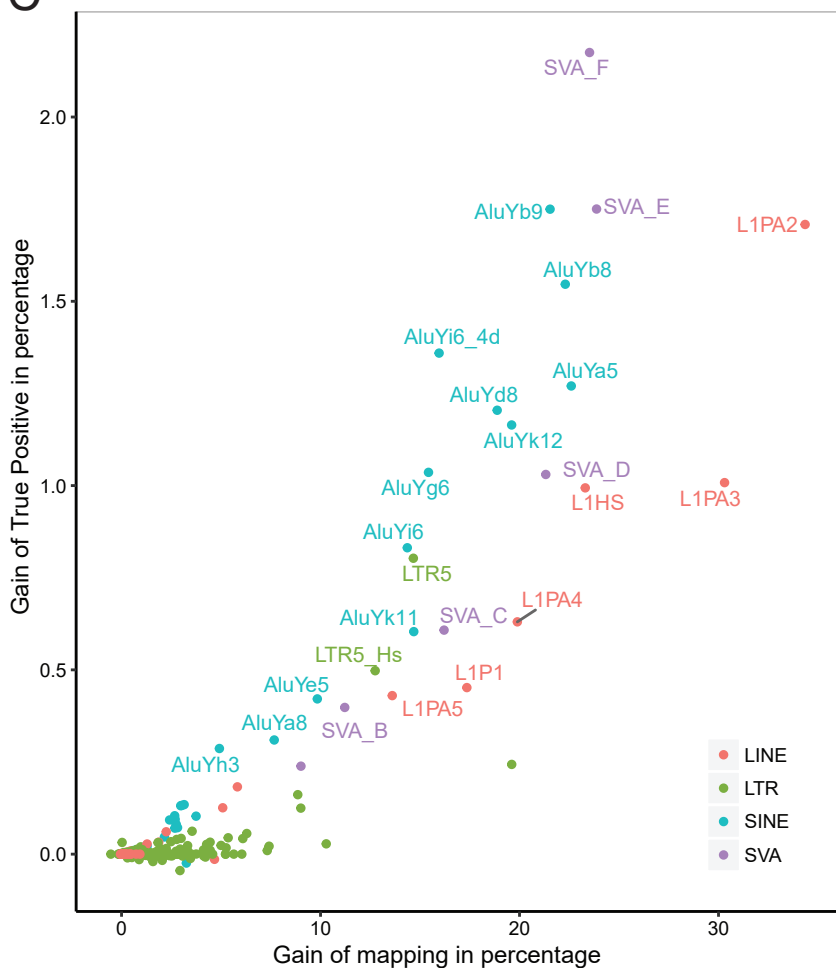

Supplement: Supplementary file 4 — Additional file 4: Figure S4. Mappability of the different human retrotransposon families. (A) True Positive (TP) rate versus mapping percentage per TE family using STAR and paired126 library and human simulated TE-derived reads. Black triangle represents the True Positive rate and percentage of mapping for the entire simulated library (B) Mapping percentage versus age of L1Md families. Dot colors represent the True Positive (TP) rate. Ages are obtained from previously published divergence analysis study (25) (C) Gain of True Positive in percentage versus gain of mapping in percentage when PE library are used in comparison to SE library. [file 13100_2019_192_MOESM4_ESM.pdf]
