# Supplementary material for: Tools and best practices for retrotransposon analysis using high-throughput sequencing data
Source: Mob DNA. 2019 Dec 29;10:52. doi: 10.1186/s13100-019-0192-1 (PMC6935493; doi:10.1186/s13100-019-0192-1)
Supplement: Supplementary file 5 — Additional file 5. Supplementary methods. [file 13100_2019_192_MOESM5_ESM.pdf]

Tools and best practices for retrotransposon analysis using high-throughput sequencing data

____

Aurélie Teissandier[1,2,3,4], Nicolas Servant[1,23,*], Emmanuel Barillot[1,2,3] and Deborah Bourc'his[1,4] *

Supplementary Methods

Mapping parameters

The following parameters were used for each mapper and mode.

Unique mode :

- Bowtie v1.0.0 : -m 1 -e 400 -v 3 --chunkmbs 100 -p 4 -I 0 -X 1000 --nomaqround -y --best –strata
- Novoalign v3.2.11 : -o SAM -r None -i 1000,200 -F STDFQ
- STAR v2.5.2b : --runThreadN 4 --outSAMtype BAM SortedByCoordinate --runMode alignReads --outFilterMultimapNmax 1 --outFilterMismatchNmax 3 --alignEndsType EndToEnd --alignIntronMax 1 --alignMatesGapMax 350
- Bowtie2 v2.1.0 : -N 1 -X 1000 -p 4 + post filtering keeping alignments when alignment score (AS tag) is higher than second valid alignment (XS tag).
- BWA aln v0.7.15 : -t 4 -n 3 + post filtering keeping alignments when XT tag is equal to U (meaning Unique alignment)
- BWA mem v0.7.15 : -t 4 -c 50000 -T 20 + post filtering keeping alignments when XT tag is equal to U (meaning Unique alignment)

Random mode :

- Bowtie v1.0.0 : -M 1 -e 400 -v 3 --chunkmbs 100 -p 4 -I 0 -X 1000 --nomaqround -y --best --strata
- Novoalign v3.2.11 : -o SAM -r Random -i 1000,200 -F STDFQ
- STAR v2.5.2b : --runThreadN 4 --outSAMtype BAM SortedByCoordinate --runMode alignReads --outFilterMultimapNmax 1000 --outSAMmultNmax 1 --outFilterMismatchNmax 3 --outMultimapperOrder Random --winAnchorMultimapNmax 1000 --alignEndsType EndToEnd --alignIntronMax 1 --alignMatesGapMax 350
- Bowtie2 v2.1.0 : -N 1 -X 1000 -p 4
- BWA aln v0.7.15 : -t 4 -n 3

- BWA mem v0.7.15 : -t 4 -c 50000 -T 20

Multi-hit mode :

- Bowtie v1.0.0 : -a -m 5000 -e 400 -v 3 --chunkmbs 100 -p 4 -I 0 -X 1000 --nomaqround -y --best --strata
- Novoalign v3.2.11 : -o SAM -r All 5000 -i 1000,200 -F STDFQ
- STAR v2.5.2b : --runThreadN 4 --outSAMtype BAM SortedByCoordinate --runMode alignReads --outFilterMultimapNmax 1000 --outFilterMismatchNmax 3 --outMultimapperOrder Random --winAnchorMultimapNmax 1000 --alignEndsType EndToEnd --alignIntronMax 1 --alignMatesGapMax 350
- Bowtie2 v2.1.0 : -N 1 -k 5000 -X 1000 -p 4

Due to complex TE content in the mouse genome, parameters were adapted using STAR when genome-wide libraries were mapped.

Mouse genome-wide mapping :

- STAR v2.5.2b unique mode : --runThreadN 4 --outSAMtype BAM Unsorted --runMode alignReads --outFilterMultimapNmax 5000 --outFilterMismatchNmax 3 --outMultimapperOrder Random --winAnchorMultimapNmax 5000 --alignEndsType EndToEnd --alignIntronMax 1 --alignMatesGapMax 350 --seedSearchStartLmax 30 --alignTranscriptsPerReadNmax 30000 --alignWindowsPerReadNmax 30000 --alignTranscriptsPerWindowNmax 300 --seedPerReadNmax 3000 --seedPerWindowNmax 300 --seedNoneLociPerWindow 1000
- STAR v2.5.2b random mode : --runThreadN 4 --outSAMtype BAM Unsorted --runMode alignReads --outFilterMultimapNmax 5000 --outSAMmultNmax 1 --outFilterMismatchNmax 3 --outMultimapperOrder Random --winAnchorMultimapNmax 5000 --alignEndsType EndToEnd --alignIntronMax 1 --alignMatesGapMax 350 --seedSearchStartLmax 30 --alignTranscriptsPerReadNmax 30000 --alignWindowsPerReadNmax 30000 --alignTranscriptsPerWindowNmax 300 --seedPerReadNmax 3000 --seedPerWindowNmax 300 --seedNoneLociPerWindow 1000
- STAR v2.5.2b multi-hit mode : --runThreadN 4 --outSAMtype BAM Unsorted --runMode alignReads --outFilterMultimapNmax 1 --outFilterMismatchNmax 3 --outMultimapperOrder Random --winAnchorMultimapNmax 5000 --alignEndsType EndToEnd --alignIntronMax 1 --alignMatesGapMax 350 --seedSearchStartLmax 30 --alignTranscriptsPerReadNmax 30000 --alignWindowsPerReadNmax 30000 --alignTranscriptsPerWindowNmax 300 --seedPerReadNmax 3000 --seedPerWindowNmax 300 --seedNoneLociPerWindow 1000

Quantification comparison

The following tools and parameters were used :

repEnrich:

bowtie --chunkmbs 10000 -p 4 -t -m 1 -S --max multimap.fastq -1 R1.fastq -2 R2.fastq > unique.sam ; samtools view –bS unique.sam > unique.bam ; samtools sort -o unique_sort.bam unique.bam ; samtools index unique_sort.bam ; RepEnrich.py rmskFile outDir nameSample genomeFolder multimap_1.fastq --fastqfile2 multimap_2.fastq unique_sort.bam --cpus 4 --pairedend TRUE --is_bed TRUE --allcountmethod TRUE

TEtools:

TEcount.py -rosette rmskFile -column 2 -TE_fasta rmskFile.fa -count TE.count -RNA R1.fastq -RNApair R2.fastq -bowtie2

TEtranscripts:

STAR --readFilesIn R1.fastq R2.fastq --runThreadN 4 --outSAMtype BAM Unsorted --runMode alignReads --outFilterMultimapNmax 100 --winAnchorMultimapNmax 100 --outFilterMismatchNmax 3 --alignEndsType EndToEnd --alignIntronMax 1 --alignMatesGapMax 350 ; samtools sort -n -o multiple_sort.bam multiple.bam ; TEtranscripts -t multiple_sort.bam -c multiple_sort.bam –TE rmsk.gtf --stranded no --format BAM –mode uniq|multi --GTF refGene.gtf --project myProject ;

SQuIRE:

squire Map -1 R1.fastq -2 R2.fastq -o outSquire -f genomeSquire -r 100 -p 4 ; squire Count -m outSquire -f genomeSquire -r 100 -p 4 -o outSquire -c genomeCleanSquire

FeatureCounts Unique Alignments:

featureCounts -F SAF -T 1 -s 0 -p -a rmsk.SAF -o outfeatureCounts.txt Input.bam

FeatureCounts Random Alignments:

featureCounts -M -F SAF -T 1 -s 0 -p -a rmsk.SAF -o outfeatureCounts.txt Input.bam

FeatureCounts Multiple Alignments:

featureCounts -M --fraction -F SAF -T 1 -s 0 -p -a rmsk.SAF -o outfeatureCounts.txt Input.bam

Supplementary Figure Legends

Supplementary Figure 1: Comparison of mapper efficiency with human simulated data. **(A)** True Positive (TP) rate versus mapping percentage with chromosome 1 of the human genome. The dots are the average values of three independent simulated libraries. SE and PE refer to single end and paired end, respectively. **(B)** Use memory, run time and size of the BAM file with chromosome 1 of the human genome. The error bars correspond to standard deviation from three independent simulated libraries.

Supplementary Figure 2: Comparison of the methods for the quantification of human retrotransposon families. **(A)** Comparison of the estimated abundance versus the true abundance for different quantification methods using human simulated TE-derived library. An R-squared value (R^2) was calculated to evaluate the correlation of estimated values between simulated values **(B)** Comparison of the estimated abundance versus the true abundance for TEtools and when randomly reported reads are used for the TE quantification with FeatureCounts (*FeatureCounts Random alignments*). A PE genome-wide library (10X coverage) was simulated using the human genome with STAR for the mapping.

Supplementary Figure 3: Impact of read depth in TE families quantification. **(A)** Estimated abundance for different quantification methods and true abundance (Simulated counts) using 5X, 10X, 25X, 50X and 100X coverage on specific mouse TE families. Only these TE families were used for the quantification. **(B)** Same as in A), with specific human TE families.

Supplementary Figure 4: Mappability of the different human retrotransposon families. **(A)** True Positive (TP) rate versus mapping percentage per TE family using STAR and paired-end library and human simulated TE-derived reads. Black triangle represents the True Positive rate and percentage of mapping for the entire simulated library **(B)** Mapping percentage versus age of L1Md families. Dot colors represent the True Positive (TP) rate. Ages are obtained from previously published divergence analysis study (25) **(C)** Gain of True Positive in percentage versus gain of mapping in percentage when PE library are used in comparison to SE library.
